# Pilot CBCT-Based Volumetric and Morphometric Analysis of the Pterygopalatine Fossa in a Romanian Cohort: A Retrospective Study

**DOI:** 10.3390/diagnostics16081182

**Published:** 2026-04-16

**Authors:** Andreea Kui, Smaranda Buduru, Anca Labunet, Simona Iacob, Elena Alexandra Iacob, Adrian Stefan Petruțiu, Dalia Popa, Iulia Valeria Ile, Marius Negucioiu

**Affiliations:** 1 Department of Prosthetic Dentistry and Dental Materials, Faculty of Dental Medicine, Iuliu Hațieganu University of Medicine and Pharmacy, 400006 Cluj-Napoca, Romania; 2 Department of Oral and Maxillofacial Surgery and Radiology, Faculty of Dental Medicine, Iuliu Hațieganu University of Medicine and Pharmacy, 400347 Cluj-Napoca, Romania

**Keywords:** pterygopalatine fossa, cone-beam computed tomography, morphometry, segmentation, volumetric analysis, maxillofacial surgery, endoscopic approaches

## Abstract

**Background:** The pterygopalatine fossa (PPF) is a complex anatomical region containing critical neurovascular structures and serving as an important surgical landmark for endoscopic and transmaxillary approaches. Quantitative data on its volumetric and linear morphology remain limited. **Objectives:** This study aimed to evaluate bilateral PPF volumes and linear dimensions using CBCT-based semi-automated segmentation, and to assess the influence of sex and age on PPF morphometry in a Romanian cohort. **Methods:** This single-center, retrospective pilot cone-beam computed tomography (CBCT) study analyzed 50 patients (28 males and 22 females; age range 16–80 years). Semi-automated segmentation was performed using ITK-SNAP software, and linear measurements were obtained from multiplanar reconstructions. **Results:** Males exhibited significantly larger mean PPF volumes than females (right: 868.96 vs. 759.86 mm³, *p* = 0.018; left: 836.71 vs. 703.00 mm³, *p* = 0.003). Significant age-related differences were observed, with younger patients (≤40 years) showing greater volumes (right: 907.06 mm³; left: 845.90 mm³) than older patients (>40 years: right: 691.80 mm³; left: 675.45 mm³; *p* < 0.0001 and *p* = 0.0003, respectively). Similar trends were observed for linear dimensions: mean height and width were consistently greater in males, and height was greater in the ≤40 age group. Left-side width did not differ significantly across age groups. No clinically relevant bilateral asymmetry was identified. Intra- and inter-observer reliability were excellent across all parameters (ICC > 0.90). **Conclusions:** These findings provide preliminary morphometric reference data on the PPF with potential implications for preoperative planning in maxillofacial surgery, endoscopic approaches, and regional anesthesia, pending prospective clinical validation.

## 1. Introduction

The pterygopalatine fossa (PPF) is a small, fat-filled, and clinically inaccessible space situated deep in the midface that functions as a major neurovascular crossroads between the nasal cavity, nasopharynx, orbit, oral cavity, masticator space, and the middle cranial fossa. Due to its intricate anatomical structure and numerous foraminal and fissural connections, the pterygopalatine fossa (PPF) often functions as a conduit for the dissemination of inflammatory processes and neoplasms among adjacent craniofacial compartments. This implies precise imaging assessment imperative for accurate diagnosis [1,2].

Cross-sectional imaging is central to the evaluation of PPF. Computed tomography (CT) is an imaging modality that provides high-resolution delineation of osseous margins and foraminal anatomy. By contrast, magnetic resonance imaging (MRI) is a technique that can depict the fat pad, neurovascular contents, and perineural tumor spread along branches of the maxillary nerve (V2). Contemporary reviews emphasize that CT and MRI are complementary and often both required to define communication pathways and disease extent across the PPF [3,4,5]. Ultra-high-field MRI has further elucidated hitherto unobserved neural intricacies within the fossa [5].

In recent decades, cone-beam CT (CBCT) has emerged as a prominent modality in maxillofacial imaging, offering several advantages over conventional methods. CBCT provides isotropic voxels, ensuring optimal spatial resolution for thin bony structures and reduced radiation exposure compared to helical CT. These attributes are particularly beneficial in the analysis of the narrow bony corridors surrounding the posterior pharyngeal wall (PPW) and its associated anatomical structures. CBCT studies have reported detailed assessments of the pterygomaxillary fissure and PPF and have begun to standardize linear and volumetric measurements relevant to surgical planning [6,7,8,9].

Beyond the initial diagnostic process, the PPF holds particular pertinence in relation to surgical and interventional procedures due to its inherent relevance in these contexts. Endoscopic endonasal and transmaxillary approaches have evolved from early proof-of-concept descriptions to mature techniques used for biopsy and the resection of benign and malignant lesions involving or traversing the posterior pharyngeal wall (PPF). These techniques frequently result in less morbidity than open approaches [10,11,12,13]. Recent comparative clinical series have refined indications and route selection for safe access to the posterior petrosal sinus and the infratemporal fossa [11,12,13].

A critical oncologic concern is perineural tumor spread (PNS) along V2 through the foramen rotundum into the PPF, which worsens prognosis and alters radiation fields and surgical strategy. Modern pictorial reviews and practice primers underscore characteristic imaging signs of PNS on CT and MRI and highlight the PPF as a pivotal waypoint along these routes [14,15]. Although the importance of the PPF in neural spread was recognized in classic CT literature [16], contemporary work has refined imaging criteria and multidisciplinary implications [14,15].

Despite the anatomical and clinical importance of the PPF, normative morphometric and volumetric reference data remain limited. A limited number of CBCT or CT studies have employed standardized landmarks and segmentation to systematically measure PPF dimensions or volume. These studies have reported factors such as laterality and sex dimorphism, yielding variable results [6,7,8,8,9,17,18]. Semiautomatic segmentation using ITK-SNAP has been proposed as a reproducible method to derive volumetric metrics from CBCT datasets; however, external validation and population-specific reference ranges are still needed [8].

ITK-SNAP (Insight Segmentation and Registration Toolkit) is an open-source multiplatform software widely used for the semi-automatic segmentation of three-dimensional medical images. Its active contour evolution algorithm, combined with manual correction capabilities, renders it particularly suitable for delineating anatomically complex structures such as the PPF, whose boundaries are defined by multiple adjacent bony walls rather than sharp density gradients. The software computes volumes directly from the segmented mask in cubic millimeters, providing a standardized, observer-independent metric that can be compared across studies adopting the same anatomical boundary definitions. Previous craniofacial morphometry studies employing ITK-SNAP have demonstrated intraclass correlation coefficients consistently above 0.85, supporting its use as a reliable platform for reproducible volumetric analysis [8]. Adoption of a common segmentation platform is a prerequisite for building cross-institutional normative databases, and the present study contributes a Romanian population dataset to this emerging reference framework.

The present study contributes to this emerging body of work by performing a CBCT-based volumetric and morphometric analysis of the PPF in a retrospective cohort. Using consistent anatomical boundaries and semiautomatic segmentation, the following is reported: (i) bilateral PPF volume and linear dimensions, (ii) differences by side and sex, and (iii) contextualization of the measurements against prior imaging studies that inform surgical corridors, anesthesia blocks, and oncologic pathways involving the PPF [6,8,8,18,19,20]. The objective of this study is to expand normative data with a reproducible workflow, with the ultimate goal of facilitating more precise preoperative planning and earlier radiologic recognition of clinically significant variation and disease spread through this critical deep facial hub.

## 2. Materials and Methods

### 2.1. Study Population

This retrospective study was based on cone-beam computed tomography (CBCT) examinations performed at the Department of Oral and Maxillofacial Surgery and Radiology, Iuliu Hațieganu University of Medicine and Pharmacy, Cluj-Napoca, Romania, between June 2021 and January 2022.

Inclusion criteria were:

- CBCT scans covering the entire pterygopalatine fossa bilaterally.
- absence of pathological conditions directly involving the fossa (e.g., tumors, cysts, vascular malformations).
- no history of surgical interventions in the midface, maxilla, or infratemporal region;
- image quality sufficient to allow accurate volumetric segmentation.

Exclusion criteria were:

- maxillofacial fractures or congenital anomalies involving the fossa;
- inflammatory or neoplastic lesions altering the region;
- motion artifacts or incomplete field of view.

All CBCT scans were anonymized before processing. The image quality criterion implicitly covers any source of artifact that compromises boundary delineation, including scatter from metallic dental restorations, though no specific metallic artifact exclusion protocol was applied, given that dental restorations rarely generate scatter extending to the depth of the PPF. Demographic characteristics and the final number of patients included are presented in the Results section.

### 2.2. Imaging Protocol

All examinations were obtained using a Planmeca ProMax 3D CBCT (Planmeca Oy, Helsinki, Finland) unit under standardized exposure settings. The voxel size was set to 0.2 mm isotropic, the field of view was set to approximately 8 × 8 cm to encompass both pterygopalatine fossae simultaneously, the tube voltage was set to 90 kV, the tube current was set to 10 mA, and the scan time was set to 15–20 s. Images were reconstructed using the standard Planmeca ProMax 3D (Planmeca Oy, Helsinki, Finland) reconstruction algorithm without additional filtering or smoothing before segmentation. The images were stored in DICOM format and exported directly to ITK-SNAP (www.itksnap.org, accessed on 1 February 2022) for analysis. At a voxel size of 0.2 mm, partial-volume effects at PPF boundaries span approximately 1–2 voxels (≤0.4 mm per boundary interface), a magnitude consistent with comparable CBCT morphometry studies [8] and mitigated by the slice-by-slice manual boundary correction performed in ITK-SNAP.

### 2.3. Volumetric Segmentation

**Figure 1:**
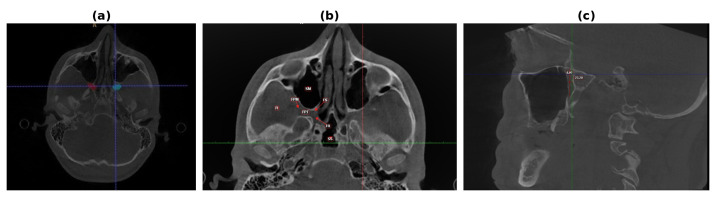
Representative CBCT images illustrating the segmentation protocol and measurement landmarks. (**a**) Axial view showing bilateral PPF segmentation masks: right fossa in red, left fossa in blue. (**b**) Sagittal view illustrating the measurement landmarks for height (superiormost to inferiormost point) and width (maximum transverse dimension). (**c**) Axial view showing bilateral PPF segmentation masks as rendered in ITK-SNAP (right fossa in red, left fossa in blue). (abbreviations in image reflect original terminology; English equivalents are provided in parentheses: FPT (PPF) = pterygopalatine fossa; FPM (PMF) = pterygomaxillary fissure; SM (MS) = maxillary sinus; FI (IT) = infratemporal fossa; FS (SF) = sphenopalatine foramen; FR = foramen rotundum; SS = sphenoid sinus)

Volumetric analysis of the pterygopalatine fossa was performed using ITK-SNAP software (version 4.0, open-source, www.itksnap.org, accessed on 1 February 2022). A semi-automatic segmentation method was employed, with manual correction when necessary. The following anatomical boundaries were used to delineate the fossa (Figure 1):

Anterior: posterior wall of the maxillary sinus;

Posterior: pterygoid process of the sphenoid bone and foramen rotundum;

Lateral: pterygomaxillary fissure;

Medial: perpendicular plate of the palatine bone; Inferior: superior opening of the greater palatine canal.

The segmentation workflow proceeded as follows: (1) DICOM data were imported and multiplanar reconstructions generated in axial, coronal, and sagittal planes; (2) a manual seed was placed within the PPF lumen on the axial view, avoiding the adjacent maxillary sinus and infratemporal fossa; (3) semi-automatic active contour evolution was performed using the ITK-SNAP default snake parameters (smoothing weight 0.2, curvature weight 0.5, advection weight 2.0) with a speed function based on the intensity gradient; (4) manual slice-by-slice boundary corrections were applied in all three planes to enforce the anatomical limits defined above; (5) volumes were automatically calculated by the software from the finalized segmentation mask and expressed in cubic millimeters (mm^3^). Manual corrections were required in approximately 60–80% of cases, primarily at the lateral boundary (pterygomaxillary fissure) and at the inferior opening of the greater palatine canal, where bony margins are least distinct. Mean segmentation time was approximately 25–35 min per patient (bilateral). Both observers performed segmentation on anonymized CBCT datasets, blinded to patient demographic data. Openings of the pterygomaxillary fissure, greater palatine canal, and vidian canal were not segmented beyond their entry points into the fossa, consistent with the boundary definitions used by Lentzen et al. [8] and Icen and Orhan [6], enabling direct cross-study comparison. The volume contributed by the included canal segments is negligible relative to the mean fossa volumes of 700–900 mm^3^.

### 2.4. Linear Measurements

In addition to volumetric assessment, linear parameters of the pterygopalatine fossa were measured on multiplanar reconstructions.

Height (the superoinferior dimension of the PPF) was measured as the distance from the superiormost to the inferiormost point of the fossa on the sagittal reconstruction. This parameter has previously been referred to as “length” in the literature; we adopt the term “height” throughout this manuscript for anatomical consistency.

The width is measured on the coronal slice that exhibits the greatest width.

The depth is measured from the anterior to the posterior boundary on the axial view.

All measurements were recorded bilaterally in millimeters (mm).

### 2.5. Reliability Assessment

All segmentation and linear measurements were performed independently by two calibrated investigators: E.A.I. (a dental medicine graduate with dedicated CBCT interpretation training) and A.K. (a specialist in prosthodontics with experience in CBCT-based morphometry). Neither investigator had access to the other’s results or to patient demographic data (sex, age) during the measurement process. Intra- and inter-observer agreement was assessed on 20 randomly selected cases, with measurements repeated by each observer after a two-week interval, using two-way mixed-effects intraclass correlation coefficients (ICC, absolute agreement).

### 2.6. Statistical Analysis

Data were compiled in Microsoft Excel (adresăMicrosoft Corp., Redmond, WA, USA) and analyzed using SPSS software (version 21, IBM Corp., Armonk, NY, USA). Descriptive statistics (mean, standard deviation, minimum, maximum) were computed for all parameters. Paired *t*-tests were used to compare right vs. left sides, while independent *t*-tests assessed differences by sex. One-way ANOVA was applied for age group comparisons. Statistical significance was set at *p* < 0.05. As this study was designed as a retrospective pilot investigation, a prospective power calculation was not performed prior to data collection. Post hoc power analysis was conducted for the primary comparisons: for the sex comparison of right-side PPF volumes (mean difference 109.1 mm^3^, pooled SD ≈ 161 mm^3^, independent *t*-test, α = 0.05, two-tailed), achieved power was approximately 0.72. For the age-group comparison (mean difference 215.3 mm^3^, pooled SD ≈ 192 mm^3^), achieved power exceeded 0.95. Primary volumetric comparisons are therefore adequately powered; linear subgroup analyses should be interpreted with appropriate caution. Given the exploratory nature of this pilot study, no correction for multiple testing was applied; all comparisons are hypothesis-generating and reported with exact *p*-values to allow independent evaluation.

### 2.7. Ethical Considerations

The study was conducted in accordance with the principles of the Declaration of Helsinki and approved by the Ethics Committee of Iuliu Hațieganu University of Medicine and Pharmacy, Cluj-Napoca (protocol code AVZ 6/16 January 2021). All patients provided written informed consent for their anonymized data to be used in this study.

## 3. Results

### 3.1. Study Population

**Figure 2:**
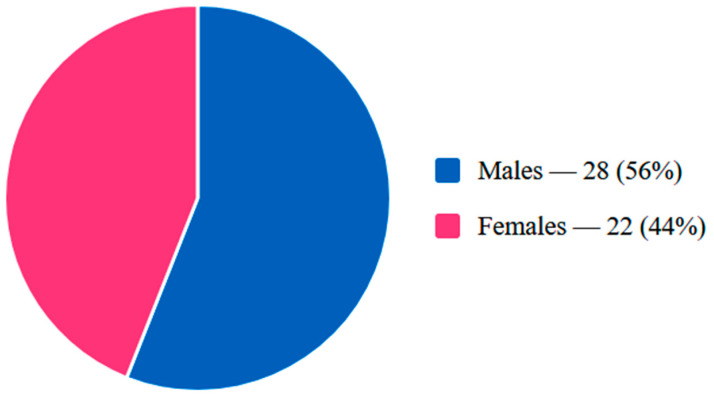
Sex distribution of the study population (*n* = 50).

A total of 50 patients were included in the study, comprising 28 males (56%) and 22 females (44%), with ages ranging from 16 to 80 years. The sex distribution is illustrated in Figure 2. When stratified by age, 30 patients (60%) were aged 40 years or younger (mean age 27.4 years), and 20 patients (40%) were older than 40 years (mean age 58.1 years). The demographic characteristics of the study population are summarized in Table 1.

### 3.2. Volumetric Analysis

In females, the mean volume of the PPF was 759.86 mm^3^ (right) and 703.00 mm^3^ (left). In males, the corresponding values were 868.96 mm^3^ (right) and 836.71 mm^3^ (left). For both sexes, the right PPF was larger than the left, with statistically significant differences (females: *p* = 0.031; males: *p* = 0.016) (Table 2).

Comparison between sexes showed that males had significantly greater volumes than females on both sides: +109.1 mm^3^ on the right (*p* = 0.018; 95% CI: 20–198 mm^3^) and +133.7 mm^3^ on the left (*p* = 0.003; 95% CI: 43–224 mm^3^).

When stratified by age, patients ≤40 years (*n* = 30) had markedly larger PPF volumes (907.06 mm^3^ right; 845.90 mm^3^ left) compared to those >40 years (*n* = 20) (691.80 mm^3^ right; 675.45 mm^3^ left). These differences were statistically significant (right: *p* < 0.0001; left: *p* = 0.0003) (Table 3).

**Figure 3:**
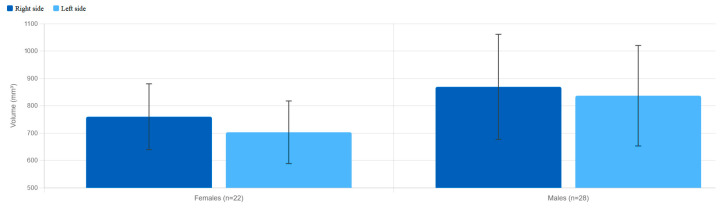
Mean PPF volumes (mm^3^) by sex and side. (Males vs. females, right vs. left. Error bars = SD).

**Figure 4:**
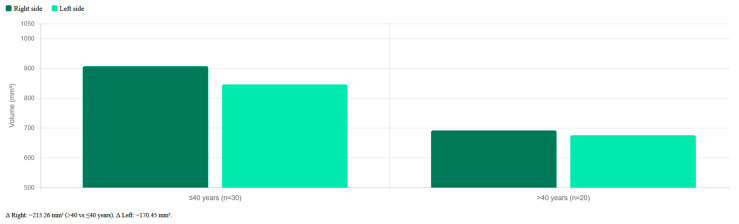
Mean PPF volumes (mm^3^) by age group (≤40 years vs. >40 years), right and left sides (∆ = difference between age groups (≤40 years vs. >40 years)).

Intra-observer reliability for volumetric measurements was excellent, with ICC values of 0.96 (right) and 0.94 (left). Inter-observer ICC values were 0.91 (right) and 0.93 (left), confirming strong agreement between the two examiners. For linear measurements, intra-observer ICC ranged from 0.91 to 0.97, and inter-observer ICC from 0.88 to 0.95 across all parameters. Figure 3 illustrates the mean PPF volumes by sex and side, and Figure 4 shows the age-group comparison. Intraclass correlation coefficients for all measured parameters are summarized in Table 4.

### 3.3. Linear Measurements

Mean height of the PPF was 20.65 mm (right) and 19.52 mm (left) in females, and 21.76 mm (right) and 21.09 mm (left) in males. Males exhibited significantly greater height values on both sides compared to females (right: +1.11 mm; left: +1.57 mm; *p* < 0.05). Mean width was 4.71 mm (right) and 4.37 mm (left) in females, and 5.34 mm (right) and 5.05 mm (left) in males. Sex differences in width were statistically significant on both sides (right: +0.63 mm; left: +0.68 mm; *p* < 0.05).

When stratified by age, patients ≤ 40 years had greater PPF height than those > 40 years on both sides (right: 22.55 vs. 19.34 mm; left: 21.52 vs. 18.74 mm; *p* < 0.05). Similarly, mean width was larger in the younger group on the right side (5.35 vs. 4.98 mm; *p* < 0.05). However, left-side width did not differ significantly between age groups (4.62 vs. 4.52 mm; *p* > 0.05), the only parameter for which the null hypothesis could not be rejected. These linear findings are consistent with the volumetric trends observed in Section 3.2 and are summarized in Table 5 and Table 6.

## 4. Discussion

### 4.1. Summary of Findings

In this retrospective CBCT study, we demonstrated that the pterygopalatine fossa (PPF) exhibits significant sex- and age-related variation. Males had larger PPF volumes than females, while patients older than 40 years showed significantly smaller fossae than younger individuals. Linear dimensions were consistently larger in males, with right–left asymmetry favoring the right side.

### 4.2. Comparison with Literature

Our results align with previous morphometric studies. Icen and Orhan reported larger volumes in males compared to females and a tendency toward right–left asymmetry using CBCT on 825 cases [6]. Liu et al. confirmed these sex-related differences in a large CT cohort [8]. Similarly, Ayaş et al. (2024) observed significant correlations between PPF size, paranasal sinus morphology, and nasal septum deviation, underlining that craniofacial anatomical variation influences PPF morphometry [18].

Kucuk (2025) reported volumetric differences in the PPF across skeletal malocclusion classes, suggesting orthodontic relevance for CBCT-based morphometry [19]. Vuksanovic-Bozaric et al. emphasized the clinical implications of PPF variability, noting the importance of preoperative imaging for endoscopic and transmaxillary approaches [20].

Our findings of reduced PPF volume with advancing age contrast with the observation by Icen and Orhan [6], who reported an age-related volume increase in a larger Turkish cohort of 825 cases. A direct methodological comparison is informative: Icen and Orhan employed continuous age modeling, used a Turkish population with potentially different craniofacial skeletal morphology, and applied boundary definitions that may differ in the treatment of the pterygomaxillary fissure. These three factors collectively account for the divergent age trends. Alhawasli et al. demonstrated that skeletal class significantly influences jaw volumetry in CBCT analyses, underscoring the importance of accounting for craniofacial skeletal variation when interpreting normative morphometric data [21]. Our binary split at 40 years cannot capture nonlinear age trajectories, and the near-significant left-side width trend (*p* = 0.18) suggests that age effects are dimension-specific. Larger prospective studies with continuous age modeling and standardized segmentation protocols are needed to clarify the direction and magnitude of age-related PPF variation across populations.

### 4.3. Clinical and Surgical Implications

Accurate morphometry of the PPF has direct implications in surgery and anesthesia. Endoscopic approaches, as reported by Bresson et al. and Plzák et al. require precise navigation around the neurovascular structures within the PPF [11,12]. Our data on sex- and age-related variability may improve surgical planning, especially for minimizing neurovascular injury.

Regional anesthesia of the maxillary nerve (V2) via the pterygopalatine approach is an established technique used in oral and maxillofacial surgery, rhinology, and for the management of cluster headache through sphenopalatine ganglion blockade. The accuracy and safety of these procedures depend critically on the three-dimensional relationship between the needle trajectory, the sphenopalatine foramen, and the posterior wall of the maxillary sinus. Our data showing that males have a significantly wider fossa (right: 5.34 vs. 4.71 mm in females) and a greater height (21.76 vs. 20.65 mm) suggest that sex- and age-related anatomical variation may be relevant to procedural planning, particularly in older female patients in whom the fossa dimensions are smallest. This observation is hypothesis-generating and requires prospective clinical correlation before specific guidance on needle trajectory or depth can be recommended.

In the context of orthognathic surgery, the pterygomaxillary fissure—the lateral boundary of the PPF—is the critical interface for the pterygoid plate separation during Le Fort I osteotomy. Variations in fossa width and depth affect the thickness of bone available at this junction and the risk of injury to the maxillary artery and its terminal branches. The age-related reduction in fossa volume documented in our study, particularly the decrease in height from a mean of 22.55 mm (≤40 years) to 19.34 mm (>40 years) on the right side, may reflect increased ossification or reduced fat pad volume, either of which could affect the tactile feedback and instrument behavior during osteotomy. These findings suggest that surgeons planning Le Fort I procedures in older patients may wish to anticipate potentially altered anatomy at the pterygomaxillary junction, pending prospective confirmation.

Patil et al. recently stressed the value of CBCT morphometry for advancing maxillofacial therapeutics, including regional anesthesia and minimally invasive approaches [22]. Furthermore, the volumetric decrease with age observed in our cohort could complicate access during interventions and may need to be considered when planning transmaxillary routes.

A specific clinical application of the morphometric data presented here concerns maxillary nerve block via the greater palatine canal approach, one of the most commonly used regional anesthesia techniques in oral and maxillofacial surgery. In this technique, a needle is inserted through the greater palatine foramen and advanced superiorly through the greater palatine canal into the pterygopalatine fossa, where the maxillary nerve (V2) is anesthetized after it traverses the foramen rotundum. The success and safety of this procedure depend critically on the superoinferior height of the PPF—the dimension along which the needle travels—and on the mediolateral width, which determines the spatial tolerance for needle deviation without neurovascular injury. Our data indicate that the mean PPF height on the right-side ranges from 19.34 mm in patients over 40 years to 22.55 mm in younger patients, a difference of over 3 mm that may require an adjustment in the insertion depth across age groups. Similarly, males exhibit a consistently wider fossa (right: 5.34 vs. 4.71 mm in females), which may influence the risk of lateral wall contact during the procedure. These observations are hypothesis-generating and require prospective clinical correlation before they can inform specific procedural guidelines.

### 4.4. Strengths and Limitations

The main strengths of our study include the use of semi-automatic segmentation with ITK-SNAP, ensuring reproducibility, and the inclusion of bilateral and sex/age-stratified analyses. Intra- and inter-observer reliability was excellent across all parameters, with ICC values exceeding 0.90 for volumes and ranging from 0.88 to 0.97 for linear dimensions, confirming the robustness of the ITK-SNAP segmentation protocol. The consistent use of a single CBCT unit under standardized acquisition settings eliminated inter-device variability, which is a recognized confounding factor in multicenter imaging studies. The bilateral design with paired analysis further strengthened the statistical comparison of right and left sides within the same individuals.

Several limitations must be acknowledged. The sample size of 50 patients, while sufficient to demonstrate significant sex and age effects, is modest for establishing population-wide normative reference ranges, and the 95% confidence intervals around the mean values should be interpreted accordingly. The single-center design limits generalizability, as craniofacial morphology is known to vary with ethnicity, geographic origin, and skeletal class. The binary age stratification at 40 years was selected to produce near-equal group sizes (*n* = 30 vs. *n* = 20) and reflects a clinically meaningful threshold between younger skeletally mature adults and older patients with progressive craniofacial remodeling. Nevertheless, it does not capture nonlinear age trajectories that continuous modeling would reveal, and future studies should employ regression-based age analysis. The achieved power for sex-based comparisons was approximately 0.72, below the 0.80 convention, consistent with the pilot nature of the study.

The absence of body-mass index, dental status, or skeletal classification data further prevents adjustment for potential confounders. Additionally, the retrospective nature of the study means that CBCT scans were originally acquired for clinical indications other than PPF assessment, which may introduce selection bias despite the rigorous inclusion and exclusion criteria applied. Finally, the study was limited to adults, and the developmental trajectory of the PPF from adolescence through skeletal maturity remains to be characterized in a dedicated pediatric cohort.

### 4.5. Future Directions

Future research should investigate PPF morphometry in pediatric and orthodontic populations, considering associations with skeletal patterns and sinonasal anatomy [18,19]. Automated segmentation with deep-learning tools, as highlighted by Zhao et al., may improve efficiency and reproducibility [23]. The integration of volumetric data into surgical navigation systems could further enhance safety and precision in PPF-related interventions.

Future studies should prospectively collect individual-level measurement data to enable the computation of medians, interquartile ranges, and Bland–Altman limits of agreement for reliability assessment, which could not be performed in the present retrospective analysis due to the absence of accessible raw measurement pairs.

## 5. Conclusions

This pilot retrospective study provides detailed volumetric and linear morphometric data on the pterygopalatine fossa (PPF) using CBCT-based semi-automatic segmentation with ITK-SNAP. The findings demonstrate that males and younger patients have significantly larger fossae compared to females and older individuals across both volumetric and linear parameters, with the exception of left-side width in the age comparison. Bilateral asymmetry was consistently present but quantitatively small and unlikely to be of direct clinical relevance in most patients. Excellent intra- and interobserver reliability confirms the feasibility and reproducibility of this segmentation workflow as a foundation for future larger studies.

The sex- and age-specific preliminary morphometric data reported here may inform preoperative planning for surgeons undertaking endoscopic endonasal or transmaxillary approaches, for anesthetists performing sphenopalatine ganglion blocks, and for radiologists interpreting perineural tumor spread through this region. The volumetric reduction observed in patients over 40 years of age—a decrease of over 200 mm^3^ on the right side compared to the younger cohort—warrants particular attention during the preoperative evaluation of older patients.

Future investigations should employ larger, multiethnic, and multicenter cohorts, integrate continuous age modeling, and correlate PPF morphometry with clinical outcomes in surgical and interventional procedures. The adoption of standardized segmentation protocols and the open sharing of normative datasets will be essential for building a robust, population-representative reference framework for this anatomically and clinically critical region.

## Figures and Tables

**Table 1:** Demographic characteristics of the study population.

Characteristic	Total (*n* = 50)	Males (*n* = 28)	Females (*n* = 22)
Age range (years)	16–80	18–80	16–76
Age ≤40 years, *n* (%)	30 (60%)	18 (64%)	12 (55%)
Age >40 years, *n* (%)	20 (40%)	10 (36%)	10 (45%)

**Table 2:** Mean volumes of the pterygopalatine fossa (mm^3^) by sex and side.

Group	Right Side (mm^3^)	Left Side (mm^3^)	*p*-Value (R vs. L)
Females (*n* = 22)	759.86 ± 120.3 (621–1102)	703.00 ± 114.5 (573–1031)	<0.05
Males (*n* = 28)	868.96 ± 192.3 (402–1171)	836.71 ± 183.8 (358–1093)	<0.05
*p*-value (M vs. F)	0.018	0.003	-

**Table 3:** Mean volumes of the pterygopalatine fossa (mm^3^) by age group.

Age Group	Right Side (mm^3^)	Left Side (mm^3^)	*p*-Value (≤40 vs. >40)
≤40 years (*n* = 30)	907.06	845.90	-
>40 years (*n* = 20)	691.80	675.45	-
*p*-value	<0.0001	0.0003	-

**Table 4:** Intraclass correlation coefficients (ICC) for intra- and inter-observer reliability of volumetric and linear measurements. Values for volume represent exact ICC estimates; values for height and width represent the observed range across right and left sides. All ICC values exceed 0.88, indicating excellent reliability. ICC = intraclass correlation coefficient.

Parameter	Intra-Observer ICC (Right)	Intra-Observer ICC (Left)	Inter-Observer ICC (Right)	Inter-Observer ICC (Left)
Volume (mm^3^)	0.96	0.94	0.91	0.93
Height (mm)	0.91–0.97 *	0.91–0.97 *	0.88–0.95 *	0.88–0.95 *
Width (mm)	0.91–0.97 *	0.91–0.97 *	0.88–0.95 *	0.88–0.95 *

* Range reported across right and left sides.

**Table 5:** Mean linear dimensions (mm) of the pterygopalatine fossa by sex and side.

Group	Height R (mm)	Height L (mm)	Width R (mm)	Width L (mm)	*p*-Value (M vs. F)
Females (*n* = 22)	20.65	19.52	4.71	4.37	—
Males (*n* = 28)	21.76	21.09	5.34	5.05	—
*p*-value (M vs. F)	<0.05	<0.05	<0.05	<0.05	—

**Table 6:** Mean linear dimensions (mm) of the pterygopalatine fossa by age group.

Age Group	Height R (mm)	Height L (mm)	Width R (mm)	Width L (mm)	*p*-Value
≤40 years (*n* = 30)	22.55	21.52	5.35	4.62	—
>40 years (*n* = 20)	19.34	18.74	4.98	4.52	—
*p*-value	<0.05	<0.05	<0.05	0.18	—

R = right; L = left.

## Data Availability

The original contributions presented in this study are included in the article. Further inquiries can be directed to the corresponding author.

## Abbreviations

The following abbreviations are used in this manuscript:

PPF	Pterygopalatine fossa
CBCT	Cone-beam computed tomography
CT	Computed tomography
ICC	Intraclass correlation coefficient
MRI	Magnetic resonance imaging
ITK-SNAP	Open-source image segmentation software (Insight Toolkit–Segmentation and Registration Toolkit)
PNS	Perineural tumor spread
SPSS	Statistical Package for the Social Sciences
DICOM	Digital Imaging and Communications in Medicine
